# Main messages for primary care from the 2016 European Guidelines on cardiovascular disease prevention in clinical practice

**DOI:** 10.1080/13814788.2017.1398320

**Published:** 2017-11-23

**Authors:** Massimo F. Piepoli, Arno W. Hoes, Carlos Brotons, Richard F.D. Hobbs, Ugo Corra, on behalf of the Task Force for the 2016 guidelines on cardiovascular disease prevention in clinical practice

**Affiliations:** ^a^ Heart Failure Unit, Cardiac Department, G Da Saliceto Hospital Piacenza Italy; ^b^ Julius Center for Health Sciences and Primary Care, University Medical Centre Utrecht Utrecht The Netherlands; ^c^ Sant Pau Institute of Biomedical Research, Research Unit, Sardenya Primary Health Care Centre Barcelona Spain; ^d^ Nuffield Department of Primary Care Health Sciences, University of Oxford Oxford UK; ^e^ Cardiology Division, Istituti Clinici Scientifici Maugeri, Centro Medico di Riabilitazione di Veruno Novara Italy

**Keywords:** Cardiovascular prevention, clinical practice, guidelines

## Abstract

In 2016, a new version of the European Guidelines on Cardiovascular Prevention was released, representing a partnership between the European Association for Cardiovascular Prevention and Rehabilitation of the European Society of Cardiology (ESC) and nine European societies, including Wonca-Europe. The ESC guidelines underscore the importance of a lifetime approach to cardiovascular (CV) risk since both CV risk and prevention are dynamic and continuous as patients’ age and/or accumulate co-morbidities. Healthy people of all ages should be encouraged to adopt a healthy lifestyle, as well as improved lifestyle and reduced risk factor levels are paramount in patients at increased risk of developing cardiovascular disease (CVD) and in those with established CVD. Healthcare professionals, and especially general practitioners, play an important role in helping patients achieve this and should set a personal example of healthy lifestyle behaviour. The ESC guidelines are based on ‘to do’ and ‘not to do’ messages. Of note, what remains uncertain is stated at the end of each dedicated chapter, confirming that guidelines are not absolute rules, and should be interpreted in the light of the healthcare worker’s knowledge and experience, patient preferences and the local social, cultural and economic situation.

KEY MESSAGES

- A lifetime approach to cardiovascular risk is underscored, since both risk and prevention are dynamic and continuous as patients’ age and/or accumulate co-morbidities.
- A great emphasis is placed on a population-based approach, on disease-specific interventions and specific subgroups deemed at increased risk such young and older individuals, women, ethnic minorities.

## Introduction

The Sixth Joint Task Force of the European Society of Cardiology and other societies on Cardiovascular Disease Prevention in Clinical Practice Guidelines were published in 2016 under the Chairmanship of Piepoli and Hoes [1]. The full text is available free as a pdf on http://www.escardio.org. The guidelines represent the product of a partnership between the main contributor, (the European Association for Cardiovascular Prevention and Rehabilitation of the European Society of Cardiology) and nine other European societies (European Association for the Study of Diabetes; European Atherosclerosis Society; European Heart Network; European Society of Hypertension; European Stroke Organisation; International Diabetes Federation European Region; International Federation of Sport Medicine; International Society of Behavioural Medicine and Wonca Europe). The partnership is based on an understanding that the partners will endeavour to ensure compatibility between the Joint Guidelines and the more specialized guidelines of the participating organizations.

The ESC guidelines on cardiovascular (CV) prevention are relevant for general practitioners, as primary care plays a crucial role in when and how to assess risk, typically by using the available risk estimation charts and risk modifiers. In addition, primary care will be instrumental in achieving risk factor targets that are outlined in the guidelines and in considering how other clinical conditions may affect cardiovascular risk estimation and management.

## 2016 European guidelines on cardiovascular disease prevention in clinical practice

Compared with the previous guidelines [2,3], greater emphasis has been placed on a population-based approach, on disease-specific interventions and specific subgroups deemed at increased risk, such as women with some female-specific conditions and certain ethnic minorities [1]. The guidelines underscore that a lifetime approach to CV risk is important since CV risk accelerates as patients’ age and they accumulate co-morbidities. Apart from improving lifestyle and reducing risk factor levels in patients with established cardiovascular disease (CVD) and those at increased risk of developing CVD, healthy people of all ages should be encouraged to adopt a healthy lifestyle. Healthcare professionals, especially those in primary care, play an important role in achieving this in their clinical practice and should set an example of healthy lifestyle behaviour.

The article is based on ‘to do’ and ‘not to do’ messages which form a summary at the end of the full text, with occasional additional information or comments. Moreover, what is unknown yet is also stated at the end of each dedicated chapter, confirming that guidelines are recommendations to be interpreted in the light of the healthcare worker’s knowledge and experience and the local social, cultural and economic situation.

## Recommendations for cardiovascular risk assessment

The 2016 guidelines recommend the assessment of total CVD risk and the prevention of CVD in an individual to be adapted to his or her total CV risk (Table 1): the higher the risk, the more intense the action should be [1]. Nonetheless, risk is a continuum and there is no exact point above which, for example, a drug is automatically indicated nor below which lifestyle advice may not usefully be offered. It is problematic to define decisional thresholds. Although individuals at the highest levels of risk gain the most from risk factor interventions, most deaths in a community come from those at lower levels of risk, simply because they are more numerous compared with high-risk individuals. Thus, a strategy for individuals at high risk must be complemented by public health measures to encourage a healthy lifestyle and to reduce population levels of CV risk factors.

**Table 1.** Recommendations for cardiovascular risk assessment.

Recommendations	Class of recommendation	Level of evidence
Systematic CV risk assessment is recommended in individuals at increased CV risk, i.e. with family history of premature CVD, familial hyperlipidaemia, major CV risk factors (such as smoking, high BP, DM or raised lipid levels) or comorbidities increasing CV risk.	I	C
It is recommended to repeat CV risk assessment every five years, and more often for individuals with risks close to thresholds mandating treatment.	I	C
Systematic CV risk assessment may be considered in men >40 years of age and in women >50 years of age or post-menopausal with no known CV risk factors	IIb	C
Systematic CV risk assessment in men <40 of age and women <50 years of age with no known CV risk factors is not recommended.	III	C

BP: blood pressure; CV: cardiovascular; CVD: cardiovascular disease; DM: diabetes mellitus.

## Who to CV risk assess

CV risk assessment or screening can be done opportunistically or systematically: opportunistic screening is without a predefined strategy, but is done when the opportunity arises, while systematic screening can be done in the general population as part of a screening programme or in targeted subpopulations. The 2016 guidelines recommend a systematic approach to CV risk assessment targeting populations likely to be at increased CV risk, such as those with a family history of premature CVD, familial hyperlipidaemia, major CV risk factors such as smoking, high blood pressure (BP), diabetes (DM) or raised lipid levels or comorbidities increasing CV risk. Systematic CV assessment may be considered in adult men >40 years of age and in women >50 years of age or post-menopausal with no known CV risk factors, while systematic CV risk assessment in men <40 years of age and women <50 years of age with no known CV risk factors is not recommended. Risk assessment is not a one-time event, but it should be repeated, for example, every five years [1].

## Recommendations for how to estimate cardiovascular risk

Since 2003, the ESC guidelines on CVD prevention propose the use of the SCORE system, which is based on large, representative European cohort datasets. Advantages and limitations of the SCORE risk charts are well acknowledged. A total CV risk stratification is recommended for adults >40 years of age, unless they are automatically categorized as being already at high or very high risk, based on documented CVD, DM (>40 years of age), chronic kidney disease (GFR <60 ml/min/1.73 m^2^) or a highly elevated single risk factor (Table 2; risk categories in Table 3). These people need active risk factor management.

**Table 2.** Recommendation for how to estimate cardiovascular risk.

Recommendations	Class of recommendation	Level of evidence
Total CV risk estimation, using a risk estimation system such as SCORE, is recommended for adults >40 years of age, unless they are automatically categorized as being at high-risk or very high-risk based on documented CVD, DM (>40 years of age), kidney disease or highly elevated single risk factor	I	C

CV: cardiovascular; CVD: cardiovascular disease; DM: diabetes mellitus; SCORE: systematic coronary risk estimation.

**Table 3.** Risk categories.

Very high-risk Subjects with any conditions in the right column.	•Documented CVD, clinical or unequivocal on imaging. Documented clinical CVD includes previous AMI, ACS, coronary revascularization and other arterial revascularization procedures, stroke and TIA, aortic aneurysm and PAD. Unequivocally documented CVD on imaging includes plaque on coronary angiography or carotid ultrasound. •DM with target organ damage such as proteinuria or with a major risk factor such as smoking or marked hypercholesterolaemia or marked hypertension. •Severe CKD (GFR <30 ml/min/1.73 m^2^). •A calculated SCORE ≥10%.
High-risk Subjects with any conditions in the right column.	•Markedly elevated single risk factors, in particular cholesterol >8 mmol/l (>310 mg/dl) (e.g. in familial hypercholesterolaemia) or BP ≥180/110 mmHg. •Most other people with DM (with the exception of young people with type 1 DM and without major risk factors that may be at low or moderate risk). •Moderate CKD (GFR 30–59 ml/min/1.73m^2^). •A calculated SCORE ≥5% and <10%
Moderate risk	SCORE is ≥1% and <5% at 10 years.
Low risk	SCORE <1%

BP: blood pressure; CVD: cardiovascular disease; DM: diabetes mellitus; SCORE: systematic coronary risk estimation; ACS: acute coronary syndrome; AMI: acute myocardial infarction; CKD: chronic kidney disease; GFR: glomerular filtration rate; PAD: peripheral artery disease; TIA: transient ischaemic attack.

In addition, there are other risk factors that could be relevant, notably when their potential to reclassify subjects to another risk category has been established. In general, reclassification is of most value when the individual’s risk lies close to a decisional threshold, such as a SCORE risk of 5%, while in (very) high-risk or low-risk situations, the impact of additional risk factors is unlikely to alter management decisions. The presence/absence of risk modifiers may move an individual’s estimated risk upward or down, since the absence of these modifiers should lead to lowering an individual’s estimated risk. Examples of modifiers are shown in Table 4.

**Table 4.** Examples of risk modifiers that are likely to have reclassification potential.

Socio-economic status, social isolation, or lack of social support.
Family history of premature CVD.
BMI and central obesity.
CT coronary calcium score.
Atherosclerotic plaques determined by carotid artery scanning.
ABI

CVD: cardiovascular disease; BMI: body mass index; CT: computed tomography; ABI: ankle–brachial blood pressure index.

Routine assessment of circulating or urinary biomarkers is not recommended for refinement of CV risk stratification. Imaging such as CT calcium scoring or ankle–brachial index can refine risk estimation in intermediate risk subjects. Carotid ultrasound intima-media thickness (IMT) screening for CV risk assessment is not currently recommended.

Due to CV risk assessment, four categories of risk have been defined (Table 2): (i) low-risk that is SCORE <1% at 10 years, (ii) moderate risk as expressed by a calculated SCORE ≥1 and <5%, (iii) high-risk, with a SCORE ≥5 and <10% and, (iv) very high-risk with a calculated SCORE ≥10% at 10 years [1]. Low to moderate-risk persons (SCORE <5%) should be offered lifestyle advice to maintain their low to moderate risk status. High-risk persons (SCORE ≥5% <10%) qualify for intensive lifestyle advice, and may be candidates for drug treatment, while in very high-risk persons (SCORE ≥10%) drug treatment is more frequently required.

## Recommendations for how to intervene and goals

Risk factors, goals, and target of levels are presented in the new version of the ESC on CVD prevention guidelines [1]. It is recommended for healthy adults of all ages to perform at least 150 minutes a week of moderate intensity or 75 minutes a week of vigorous intensity aerobic physical activity (PA) or an equivalent combination thereof. PA is recommended in low risk individuals without further assessment. Avoidance of exposure to any form of tobacco is recommended. Smokers should be offered repeated advice on stopping with offers to help aided by the use of follow up support, or therapies. A healthy diet low in saturated fat with a focus on wholegrain products, vegetables, fruit and fish is recommended as a cornerstone of CVD prevention in all individuals. It is recommended that subjects with healthy weight maintain their weight and that overweight and obese people aim for a progressive reduction in weight to a BMI of 20–25 kg/m^2^ or a waist circumference of <94 cm (men) or <80 cm (women).

In very high CV risk subjects, an LDL-C goal <1.8 mmol/l (<70 mg/dl), or a reduction of at least 50% if the baseline is between 1.8 and 3.5 mmol/l (70 and 135 mg/dl) is recommended, while in high CV risk people, an LDL-C goal <2.6 mmol/l (<100 mg/dl), or a reduction of at least 50% if the baseline is between 2.6 and 5.1 mmol/l (100 and 200 mg/dl) is recommended. In most of patients, this will require treatment since diet will produce at most a 6% reduction in LDL. The most evidence-based therapies for lipid lowering are statins, which reduce all CV events by around 23% per mmol reduction in LDL. Statins are very effective agents but are not always tolerated. Adverse effects include mainly muscle symptoms, but the risks of side effects are often over-stated. The only other treatments with known effectiveness are ezetimibe or the new, very expensive, PCSK9 inhibitors.

In treated hypertensive patients <60 years old, systolic blood pressure (SBP) < 140 mmHg and diastolic blood pressure (DBP) < 90 mmHg are preventive goals, and in patients >60 years old with SBP ≥160 mmHg, it is recommended to reduce SBP to between 150 and 140 mmHg. In individuals >80 years and with initial SBP ≥160 mmHg, it is recommended to reduce SBP to between 150 and 140 mmHg, while BP target is <140/85 mmHg in type 2 DM. A BP <130/80 mmHg is recommended in selected patients (e.g. younger patients at elevated risk for complications), and BP target in patients with type 1 DM is <130/80 mmHg. Drug treatment is recommended in patients with grade three hypertension (BP 180/110 mmHg or higher) irrespective of CV risk (as well as in patients with grade one (BP 140–159/90–99) or two (BP 160–179/100–109) hypertension who are at very high CV risk. All major BP lowering drug classes (i.e. diuretics, ACE-I, calcium antagonists, ARBs, and ß-blockers) do not differ in their BP-lowering effect. A renin-angiotensin-aldosterone system blocker is recommended in the treatment of hypertension in DM, particularly in the presence of proteinuria or micro-albuminuria. ß-blockers and thiazide diuretics are not recommended in hypertensive patients with multiple metabolic risk factors. Anti-hypertensive drugs to be preferred in specific conditions are listed in Table 5.

**Table 5.** Drugs to be preferred in specific conditions.

Condition	Drug
Asymptomatic organ damage	
LVH	ACE-I, calcium antagonist, ARB.
Asymptomatic atherosclerosis	ACE-I, calcium antagonist.
Microalbuminuria	ACE-I, ARB.
Renal dysfunction	ACE-I, ARB.
Clinical CV event	
Previous stroke	Any agent effectively lowering BP.
Previous MI	β-blockers, ACE-I, ARB.
Angina pectoris	β-blockers, calcium antagonist.
Heart failure	Diuretic, β-blockers, ACE-I, ARB, mineralocorticoid receptor antagonist.
Aortic aneurism	β-blockers.
AF: prevention	*Consider* ARB, ACE-I, β-blockers or mineralocorticoid receptor antagonist.
AF: rate control	β-blockers and no-dydropyridine calcium antagonist.
ESRD/proteinuria	ACE-I, ARB.
PAD	ACE-I, calcium antagonist.
Other condition	
ISH (elderly)	Diuretic, calcium antagonist.
DM	ACE-I, ARB.
Pregnancy	Methyldopa, β-blockers, calcium antagonist.
Black people	Diuretic, calcium antagonist.

BP: blood pressure; CV: cardiovascular; DM: diabetes mellitus; PAD: peripheral artery disease; ACE-I: angiotensin-converting enzyme inhibitor; ARB: angiotensin receptor blocker; AF: atrial fibrillation; ISH: isolated systolic hypertension; LVH: left ventricular hypertrophy; MI: myocardial infarction.

A target HbA1c for the reduction in risk of CVD and microvascular complications in DM of <7.0% (<53 mmol/mol) is recommended for the majority of non-pregnant adults with either type 1 or type 2 DM. In DM, metformin is recommended as therapy, if tolerated and not contra-indicated, following evaluation of renal function. Lipid-lowering agents (principally statins) are recommended to reduce CV risk in all patients with type 2 or type 1 DM above the age of 40 years.

Finally, antiplatelet therapy is not recommended in individuals without CVD due to the increased risk of major bleeding.

## Recommendations for achieving medication and healthy lifestyle adherence

Simplifying the treatment regimen to the lowest acceptable level is recommended, with repetitive monitoring and feedback. In the case of persistent non-adherence, multiple sessions combined with behavioural interventions if feasible are recommended. It is recommended that health personnel and caregivers set an example by following healthy lifestyle, such as not smoking or using tobacco products at work.

## Recommendations for how to intervene at the individual level: disease-specific interventions

Prevention strategies should be started as soon as possible, and the commitment of physicians dealing with acute CV events is crucial. Moreover, an appropriate discharge phase linking acute and chronic care to promote preventive strategies and maintain preventive goals in the long-term are suggested.

## Recommendations for how to intervene at the population level

The population level approach follows the Geoffrey Rose paradigm: small shifts in the risk of disease (or risk factor) across a whole population consistently lead to greater reductions in disease burden than a large shift in high-risk individuals only. A population-wide approach has further advantages, as it addresses CV health over the entire life course and reduces health inequalities. Several key messages are provided according to risk factors in the general population, but cost-effectiveness, sustainability and scientific background of recommendations are not available.

## Recommendation for CVD prevention implementation

Preventive programmes for therapy optimisation, adherence and risk factor management are recommended in CVD patients to reduce disease recurrence and in high-risk patients without CVD to prevent CVD occurrence. The role of primary care is highlighted. It is recommended that general practitioners, nurses and allied health professionals within primary care work as a team to deliver CVD prevention for high-risk patients. Nurses and allied health-professional led programmes have been shown to be effective in CVD prevention across healthcare settings. Methods to increase referral to and uptake of cardiac rehabilitation should be considered such as electronic prompts or automatic referrals, referral and liaison visits, structured follow-up by physicians, nurses or therapists, and early starts to programmes after discharge.

In the acute hospital setting, it is recommended to implement strategies for prevention in CVD patients, including lifestyle changes, risk factor management and pharmacological optimization, after an acute event before hospital discharge. Participation in a cardiac rehabilitation programme for patients hospitalized for an acute coronary event or revascularization, and for patients with heart failure is recommended.

## Summary overview of the most relevant changes

- A strategy for individuals at high risk is complemented by public health measures to encourage a healthy lifestyle and to reduce population levels of CV risk factors. A combined strategy is advocated to improve CV health across the population at large from childhood onwards, with specific actions to improve CV health in individuals at increased risk of CVD or with established CVD.
- Specific chapters are dedicated to a population-based approach to promote healthy environment and healthy lifestyle, including diet, exercise, smoking cessation, avoidance of alcohol abuse. Healthcare professionals have an important role as advocates of this approach.
- There is more emphasis on CV risk markers in relevant groups, such as young and older individuals, women, and ethnic minorities.
- Promotion of physical activity and healthy lifestyle in all settings and in all population groups, starting in childhood.
- Health personnel, caregivers should set an example by following healthy lifestyle, such as not smoking or using tobacco products.
- Specific sections on preventive interventions at the individual level are included for patients with specific diseases, including atrial fibrillation, coronary artery disease, chronic heart failure, cerebrovascular disease, peripheral artery disease.
- Monitoring the process of delivery of CVD prevention activities and outcomes is considered.
